# Sexuality and Well-Being Among Community-Dwelling Older Adults in Bogotá, Colombia

**DOI:** 10.1093/geroni/igaf122.2775

**Published:** 2025-12-31

**Authors:** Maria Reyes, Natalie Levy, Maria-Jose Castro, Gabriela Morales, Gabriela Garcia, Maria Ordoñez, Angie Riveros

**Affiliations:** Universidad de los Andes, Bogota, Distrito Capital de Bogota, Colombia; Universidad de los Andes, Bogota, Distrito Capital de Bogota, Colombia; Universidad de los Andes, Bogota, Distrito Capital de Bogota, Colombia; Universidad de los Andes, Bogota, Distrito Capital de Bogota, Colombia; Universidad de los Andes, Bogota, Distrito Capital de Bogota, Colombia; Universidad de los Andes, Bogota, Distrito Capital de Bogota, Colombia; Universidad de los Andes, Bogota, Distrito Capital de Bogota, Colombia

## Abstract

Sexuality in late life is often overlooked, yet it plays a significant role in the well-being and mental health of older adults. We conducted a mixed methods study to explore the relationship between sexuality, well-being, and psychosocial factors among older adults in Bogotá, Colombia. The quantitative phase included 615 older adults (Mage= 66), 57.3% women, 40.9% men, and 1.4% transwomen. Surveys measured sexual well-being, depression, resilience, life satisfaction, and social support. The qualitative phase involved interviews with 19 participants (7 women, 6 men, and 6 transwomen), exploring experiences of sexuality, intimacy, and interactions with healthcare providers.

**Results:**

multiple linear regression analysis showed that overall sexual satisfaction was predicted by age and awareness of the right to a fulfilling sexual life. Significant correlations were found between higher satisfaction with social support and perceived sexual compatibility and overall sexual satisfaction. Low social support was associated with higher distress in sexual functioning. Qualitative findings highlighted stigma surrounding aging and sexuality, particularly among transgender individuals. Participants highlighted the continued relevance of sexuality, especially for those in active relationships. While some saw no changes with aging, most reported significant shifts. Participants revealed that professionals rarely addressed sexuality.

**Discussion:**

social support, awareness of sexual rights, and resilience were positively associated with higher sexual satisfaction. Participants recognized changes in their sexuality as they aged. Cultural and religious beliefs played a crucial role in discussing sexuality, as many participants initially felt barriers to speaking openly. However, they acknowledged that sexuality is an important yet hidden topic.

